# Dwellers and Trespassers: Mononuclear Phagocytes at the Borders of the Central Nervous System

**DOI:** 10.3389/fimmu.2020.609921

**Published:** 2021-03-05

**Authors:** Daniela C. Ivan, Sabrina Walthert, Kristina Berve, Jasmin Steudler, Giuseppe Locatelli

**Affiliations:** Theodor Kocher Institute, University Bern, Bern, Switzerland

**Keywords:** macrophage cell, meninges, CNS inflammation, cell trafficking, choroid plexus

## Abstract

The central nervous system (CNS) parenchyma is enclosed and protected by a multilayered system of cellular and acellular barriers, functionally separating glia and neurons from peripheral circulation and blood-borne immune cells. Populating these borders as dynamic observers, CNS-resident macrophages contribute to organ homeostasis. Upon autoimmune, traumatic or neurodegenerative inflammation, these phagocytes start playing additional roles as immune regulators contributing to disease evolution. At the same time, pathological CNS conditions drive the migration and recruitment of blood-borne monocyte-derived cells across distinct local gateways. This invasion process drastically increases border complexity and can lead to parenchymal infiltration of blood-borne phagocytes playing a direct role both in damage and in tissue repair. While recent studies and technical advancements have highlighted the extreme heterogeneity of these resident and CNS-invading cells, both the compartment-specific mechanism of invasion and the functional specification of intruding and resident cells remain unclear. This review illustrates the complexity of mononuclear phagocytes at CNS interfaces, indicating how further studies of CNS border dynamics are crucially needed to shed light on local and systemic regulation of CNS functions and dysfunctions.

## Introduction

The borders of the central nervous system (CNS) parenchyma are complex structures which maintain organ homeostasis through distinct anatomical specializations. These border areas halt the transit of potentially harmful trespassers contributing to the establishment of a relatively immune-privileged milieu within the CNS parenchyma (1). At the same time, these functional barriers host an extensive variety of yolk sac- and bone marrow-derived myeloid cells, cellular dwellers which are an integral part of the historically overlooked CNS immune capabilities. Altogether, CNS interfaces are fundamental participants in CNS functions and defense mechanisms, as well as contributing to the overall integration of the CNS with the rest of the organism (2–4).

While an increasing body of research is finally dedicating attention to CNS borders and their cellular components, surprisingly much remains to be investigated and understood (5–7).

In this review, we will illustrate the functions and migratory routes of monocyte-derived and tissue-resident macrophages, the immune cells that most densely populate CNS interfaces during homeostasis and upon damage and inflammation (8).

## Barrier-Associated Dwellers: Location and Homeostatic Function of CNS Macrophages

CNS borders contain functional barriers separating the CNS parenchyma from peripheral circulation at the level of I- the leptomeningeal/subpial vasculature within the subarachnoid space (SAS), II- the blood-cerebrospinal fluid barrier (BCSFB) of the choroid plexus (ChP) and of the arachnoid mater, and III- the blood-brain/spinal cord barrier (BBB) within parenchymal vessels. As an exception to this rule, circumventricular organs lining the brain ventricles and possessing endocrine functions lack a BBB (9). Furthermore, the CNS parenchyma is protected by the astrocytic glia limitans which envelops perivascular and meningeal surfaces (10) allowing a double layer of separation between parenchymal cells and peripheral circulation (11–13).

**Figure 1:**
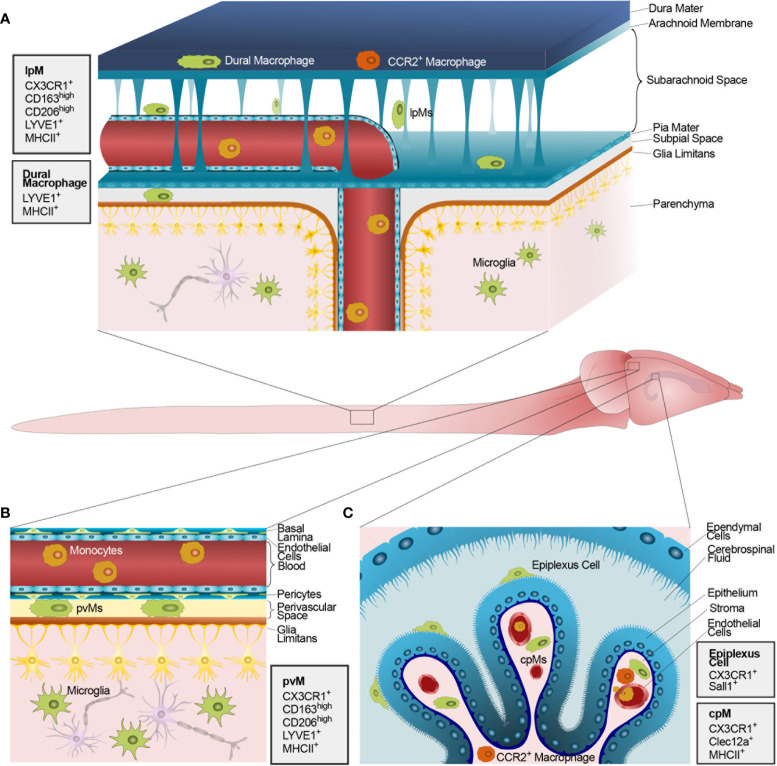
Macrophages populating the CNS barriers and parenchyma at steady state. The figure shows the mouse CNS and, in the magnified inlets, schematic representations of the anatomical CNS interfaces containing functional barriers. **(A)** The mouse meninges including (top to bottom) the dura mater, the impermeable arachnoid mater, the SAS, the pia mater, the astrocytic glia limitans, and, finally, the CNS parenchyma. The dura mater is populated by both yolk sac-derived (green) and blood-borne CCR2^+^ macrophages (orange). Conversely, the SAS, the subpial space and the CNS parenchyma host solely long-lived yolk sac-derived lpMs and microglia, respectively. **(B)** Schematic representation of the perivascular space at the level of post-capillary venules within the CNS parenchyma. The perivascular space hosts yolk sac-derived pvMs between a layer composed of endothelial basal lamina and pericytes and a parenchymal basal lamina. Endothelial cells forming the blood vessel are linked by tight junctions thus constituting a BBB. On the parenchymal side, astrocytic end-feet collectively form the glia limitans vascularis. **(C)** Schematic representation of the ChP within a CSF-filled brain ventricle lined by ependymal cells. On the apical side of the ChP epithelial cells, resident epiplexus cells are shown. ChP epithelial cells are linked by tight junction thus constituting a functional BCSFB. The ChP stroma hosts a combination of yolk sac-derived cpMs (green) and blood-borne CCR2^+^ macrophages (orange) extravasated from stromal vessels lacking a BBB. Monocytes circulating within vascular lumens are shown in yellow. Next to each panel, gray boxes illustrate the main protein markers identifying CNS-resident macrophages in their distinct anatomical compartments.

CNS interfaces harbor populations of tissue-resident macrophages often referred to as CNS-associated macrophages or barrier-associated macrophages (BAMs, **Figure 1**) (14–16). Once mistakenly believed to derive from adult bone marrow progenitors (17, 18), most BAMs originate in the yolk sac during embryonic development and stably populate the respective niches by self-renewal throughout adulthood (14), as previously shown for microglia (19). The complex development of BAMs and microglia, deriving from distinct yolk sac-derived progenitor lineages (20), has been extensively reviewed in the last years (7, 21). Sharing high expression of fractalkine receptor (CX3CR1) and a long half-life, compared to circulating monocytes, BAMs and microglia have been often collectively studied as CNS-resident phagocytes (22), at least until the recent discovery of microglia-specific genes and related targeted transgenic approaches (15, 23).

Compared to microglia, BAMs share universally upregulated genes linked to blood vessel development, lipid and cholesterol metabolism, immune response and antigen presentation (16). In addition to the core genes *Apoe*, *Ms4a7*, *Ms4a6c*, *Tgfbi* and *Mrc1* (16), *Dab2*, *F13a1*, *Mgl2*, and *Pf4* have been recently proposed as BAM identifiers (24).

Not surprisingly, BAMs also express signature macrophage markers such as integrin αM (CD11b), Aif1 (Iba1), receptor for macrophage-colony stimulating factor (Csf1R), and F4/80 (25), the latter, however, at lower levels compared to activated macrophages and circulating monocytes (26). Expression of the adhesion molecule CD44 is negligible and can thus be used to distinguish BAMs from CD44^+^ blood-borne macrophages within the CNS (26). Interestingly, some BAMs express the gene encoding for the T cell receptor β, although its function remains unknown (26).

While BAMs at the BBB and within the leptomeninges are solely yolk sac-derived, dura mater, and ChP interfaces harbor a mixed resident population including blood-borne monocyte-derived cells during steady state (14, 16). Novel techniques such as mass cytometry (through CyTOF) and single-cell RNA sequencing (scRNAseq) have indeed revealed a surprising heterogeneity of BAMs (16, 26–28), despite the intrinsic limitations of these approaches due to the use of predefined markers (mass cytometry) and under-representation of lowly expressed genes (scRNAseq) (16, 29).

In the next chapters, we will illustrate how BAM complexity is inherently linked to the different anatomical locations that these cells inhabit (30). A summary of cellular locations, origin and known markers in mice and humans can be found in **Tables 1** and **2**.

**Table 1:** The table indicates the main RNA and protein markers described for macrophage populations in the distinct CNS compartments in mice at steady state (homeostasis) and in different disease model.

Murine Models	Circulating Monocytes	BAMs (general markers)	lpMs	pvMs	cpMs	MdMs
**Location** →	**Blood**	**CNS borders**	**Leptomeninges**	**Perivascular Spaces**	**Choroid Plexus**	**CNS**
**Origin** →	**Bone Marrow**	**Yolk Sac**	**Yolk Sac**	**Yolk Sac**	**Bone Marrow/Yolk Sac**	**Bone Marrow**
**Homeostasis**	Inflammatory cells: LY6C^high^ CCR2^+^ CX3CR1^low^ (31) Patrolling cells: LY6C^low^ CCR2^low^ CX3CR1^high^ (31) Shared markers: CSF1R, GM-CSFR, PECAM-1, β2, αM integrins (32)	*Apoe*, *Ms4af*, *MS4a6c*, *Tgfbi*, *Mrc1* (16) *Dab2*, *F13a1*, *Mgl2*, *Pf4* (24) CX3CR1 (27) CD11b, IBA1, CSF1R, F4/80 (25)	*Pf4*, *Cbr2*, *Ms4a7*, *Stab1*, *Fcrls*, *Siglec1* (27) *P2rx*, *Egfl7*, *Clec4n*, *Clec10a*, *Folr2*, *Lyve1* (16) Certain populations: *Cxcl2*, *Nfkbiz* (27) CD163^high^ CD206^high^ (33–34) SAS LYVE1^low^MHCII^high^ Pial LYVE1^high^MHCII^low^ (15) CX3CR1^low^ LYVE1^+^ CD38^+^ (15, 16)	*Mrc1*, *Ms4a7*, *Cbr2*, *Pf4*, *Stab1*, *Lyve1* (27) MHC-II (35) CD163^high^ (35) CD36 (14, 36) CD38 (15)	*Mrc1*, *Ms4a7*, *Pf4*, *Stab1*, *Cbr2*, *Fcrl* (27) *Lilra5*, *Ttr* (16) Kolmer´s Epiplexus Ms: *Sall1*, *Cst7*, *Gm1673*, *Clec7a* (16) LYVE1^+^ MHCII ^negative^ LYVE1^negativ e^MHCII^+^ LYVE1^+^ MHCII^+^ (15) CCR2 (16, 37) CD163^+^MHCII^+^ (38, 39) Bone marrow derived resident Ms: MHCII^low^ Yolk sac derived resident Ms: MHCII^high^ (16)	
			**Dural Ms**			
			**Yolk Sac and Bone Marrow**			
			Lyve1^low^MHCII^high^ (majority) Lyve1^low^MHCII^+^ (minority) (15)			
**EAE**		CD11b, CSF1R, CD163, CD206 (21) MHCII, CD44, PDL1, CD117, SCA-1 (15)	*Ccl5*, *H2-Ab1*, *H2-Aa*, *H2-Eb1*, *Cd74* (27) LYVE-1 (27) IBA1^high^ (40)	*Ccl5* ^high^ *Cd74* ^high^ *Lyve1* ^low^ *Ctsd* ^low^ (27, 41) OX6, SILK6, CD40, CD80, CD86 ICAM-1, VCAM-1, CCL2, CCL3 (42)	*Ctss*, *Il1β*, *S100a9*, *S100a8*, *Ngp* (27)	*Mrc1*, *Fn1*, *Cd44*, *Mertk*, *Cd206* (27) *Saa3+*, *Cxcl10+* (43) *C1qa*, *C1qc* (44) CCR2^+^ Ly6C^high^ (44) MMP2, MMP9 (45, 46) CD44 (26) iNOS, Arginase-1 (44) F4/80^high^ (26) ChP MdMs: *Cd209*, *MertK* (27) CD74, LY6C^high^ (27), CCR2 (37)
**TBI/SCI Models**	LY6C^hi^ CX3CR1^low^CCR2^hi^ LY6C^lo^ CX3CR1^high^CCR2^lo^ (47)		LYVE1^+^ (48)	LYVE1^+^ (48)		CD163^+^ HO-1^+^ (49) CCR2^+^ (50)
**PD Models**	CCR2^+^ (51)			CD206^+^ (52)		CD163^+^ (53)
**AD Models**	CX3CR1^+^LY6C^low^ (54)			SR-B1 (55) CCR2 (56) CD36 (57)	TREM2^+^ (58)	CD45^high^ CD11b^high^ CCR2^+^ (59)

**Table 2:** The table indicates the main RNA and protein markers described for macrophage populations in the distinct CNS compartments in human samples at steady state (homeostasis) and upon development of different CNS pathologies.

Human	Circulating Monocytes	BAMs (general markers)	lpMs	pvMs	cpMs	MdMs
**Location** →	**Blood**	**CNS borders**	**Leptomeninges**	**Perivascular Spaces**	**Choroid Plexus**	**CNS**
**Homeostasis**	CD14^high^ CD16^-^ CD14^+^ CD16^high^ CD14^high^ CD16^+^ (32, 60, 61)	*Stab1*, *Ch25h* *(* 62)			Iba1^+^CD68^+^MHCII^+^ (majority) MHCII^negative^ Iba1^+^ cells (minority) (63, 64)	
**MS**	CD14^+^ CD16^high^ (65) CD14^high^CD16^high^ (66)		Yolk Sac derived: CD68+ (67) CSF monocytes (bone marrow derived) *Cd9*, *Cd163*, *Egr1*, *Btg2*, *C1qa*, *C1qb*, *Maf*, *Csf1r*, *Stab1*, *Ch25h*, *Lyve1*, *Trem2*, *Tmem119*, *Gpr34* (62) Cd16^+^ CCR5^high^ CD64^+^ CD86^+^CD14^high^ (68) HLA-G (69) HLA-DR^+^ CD33^+^ Lyve1^+^ (70) CD14+ FCGR3A/CD16^intermediate^ (62)	CD68, CD64, CD40, CD32, MHCII CD163, CD206 (71, 72)	Iba1^+^CD68^+^MHCII^+^ (majority) MHCII^negative^ Iba1^+^ cells (minority) (63, 64)	Pv MdMs: *Nrf2* (73)
**TBI**				CD163^+^ (74)		CD14^+^ (75)
**PD**			CSF monocytes: MHC-II^+^ (76)	CD206^+^ (52)		CD163^+^ (77) CCR2^+^ (78, 79)
**AD**					TREM2^+^ (80)	CD163^+^ (77)

### Resident Perivascular Macrophages

The low pinocytic endothelial cells forming parenchymal CNS vessels possess specialized features constituting the BBB, a relatively impermeable diffusion barrier (81, 82). On the parenchymal side, astrocytic end-feet form the glia limitans to offer a second functional barrier protecting the CNS parenchyma. This astrocytic layer appears impermeable to immune cells (13) but does not form tight junctions during homeostasis and allows movement of low-molecular weight tracers (83). Together, this multilayered border limits trafficking of circulating immune cells and controls the selective exclusion of harmful substances from the CNS parenchyma as well as the intake of water, chemicals, and other molecules (3).

First described in the early 1980s as “granular pericytes” (84), perivascular macrophages (pvMs) reside between the endothelial and glia limitans basement membranes of CNS vessels (excluding capillaries and small arterioles) located in basal ganglia and white matter (85–87). PvM distribution remains, however, controversial, with recent work reporting similar densities of pvMs in peri-arteriolar and peri-venous space of the mouse brain (88).

Given their strategic location, pvMs are proposed to mediate passage of information between the CNS and the periphery (4) and to regulate lymphocyte immunesurveillance (89, 90). Indeed, pvMs express MHC class II and co-stimulatory molecules (35) and secrete cytokines and chemokines, which affect the local microenvironment upon sensing damage or inflammation (8). Moreover, pvMs help to maintain the well-being of the endothelial wall and to contribute to the regulation of vascular permeability (91, 92). In line with the physiological function of the perivascular space (93), PvMs participate in CNS waste clearance (94, 95) displaying a high endocytic rate that can be exploited to mark these cells *in vivo* (94, 96–98). PvMs can also phagocytose tracers injected in the parenchyma, which demonstrates their ability to sample outflowing CNS interstitial fluids (12). Altogether, given the influence of pvMs on vascular smooth muscle cells (88) and the importance of pvMs on peri-arterial drainage (99), these cells appear key players in CNS fluid dynamics.

Morphologically, pvMs are compact elongated cells displaying continuous movement of cell body and protrusions (14, 100). Homeostatic pvMs are a transcriptionally homogeneous population (27). Compared to monocytes and microglia, pvMs are characterized by high expression of *Cd163* (35), a pattern recognition receptor (PRR) recognizing hemoglobin (101), *Mrc1* (CD206), a PRR responsible for scavenging circulating glycoproteins (102), and *Cd36*, a scavenger receptor implicated in efferocytosis (14, 36). Mass cytometry revealed that these cells, similarly to other BAMs, are also highly positive for CD38 (15), an ecto-enzyme with metabolic functions (98).

Earlier reports, likely affected by the technical challenge of distinguishing dendritic cell (DC) from BAMs (15), indicated expression of DC markers such as CD11c and DC-SIGN in pvMs (103). Functionally and ontogenically separate from BAMs, CNS-associated DCs are described elsewhere (16, 27, 28).

### Resident Leptomeningeal Macrophages

The cerebrospinal fluid (CSF)-filled SAS regulates CNS fluid, pathogen, and immune cell dynamics (104) and hosts several types of immune and non-immune dwellers including leptomeningeal macrophages (lpMs). The SAS is contained between the tight arachnoid membrane and the pia mater, a thin monolayer of cells linked by desmosomes and gap junctions (104–107). Different collagen-rich trabeculae covered by pial/leptomeningeal cells connect the arachnoid to the pia mater in humans (103). Finally, below the pia mater, the glia limitans functionally separates the SAS from the parenchyma delineating the entire CNS (10, 13).

Importantly, the CSF permeating the SAS also fills the perivascular spaces of parenchymal vessels, with complex exchanges at the level of penetrating arteries surrounded by a layer of pial cells (108). The CSF also collects antigen-rich interstitial fluid from the CNS parenchyma (106), although the extent of this process remains the subject of debate. Accordingly, intra-CNS administration of drugs or tracers [e.g., intra-ventricular injection of clodronate particles (109)] leads to targeting of both lpMs and pvMs (88, 95), an often-overlooked phenomenon in BAM literature.

Altogether, both pvMs and lpMs continuously surveil CSF composition and thus indirectly examine the CNS at a molecular level (12). Given the high local production of immune-regulatory molecules such as TGFβ2 and IL13, the CSF can also influence the phenotype of resident SAS cells (110).

Long-lived lpMs originate in the yolk sac and seed the SAS embrionically (14). Similarly to pvMs, lpMs show an impaired potential for self-renewal following drug-induced inhibition of Csf1r, at least compared to fast-proliferating microglia (16).

LpMs constitute approximately 1/3 of the cells collected from human CSF (111) but are also found in high densities in the subpial layer above the parenchyma (38). Within the SAS, they are often located nearby fibroblast-like leptomeningeal cells (14). Morphologically, lpMs have been described as sessile elongated cells following leptomeningeal vessels (100). Recent intravital observations showed, however, that lpMs are heterogenously able to remain stationary with continuous ameboid movement or to crawl within the SAS (14).

As other BAMs, lpMs are CD163^high^CD206^high^ sentinels for pathogens and inflammation (33, 34, 49) and important sources of the chemoattractant CXCL12/SDF-1, a key factor in the migration of immune cells and neuronal and oligodendrocyte precursors (112, 113). On a transcriptional level, homeostatic lpMs express high levels of *Pf4*, *Cbr2*, *Ms4a7*, *Stab1*, *Fcrls*, and *Siglec1*, with certain subpopulations expressing *Cxcl2* and *Nfkbiz* (27). A different scRNA-seq study also indicated high expression of *P2rx7*, *Egfl7*, *Clec4n*, *Clec10a*, *Folr2*, and *Lyve1*, with a comparable expression pattern from birth to adulthood (16). Among these, Lyve1, a hyaluronic acid receptor highly expressed in lymphatic vessels (114), has emerged as a marker for MHCII^low^ lpMs close to the pia mater (15), as opposed to its low expression in MHCII^high^ lpMs in the SAS (115).

Interestingly, the SAS hosts a small population of CX3CR1^low^Lyve1^+^CD38^+^ lpMs (15, 16) which might have escaped characterization in studies discriminating BAMs based on CX3CR1 positivity (14, 27).

### Resident Dural Macrophages

The dura mater is the outermost component of the meninges, containing a high density of collagen and blood vessels that lack a BBB (104, 116). Differentiating this compartment from the rest of the CNS and similar to peripheral organs, the dura displays lymphatics running along major venous sinuses (93) and thus cannot be considered a CNS immune barrier (13, 28). Furthermore, the dura remains delineated from the SAS by a functional BCSFB containing intercellular tight junctions, the impermeable arachnoid membrane (13, 104, 116). While the possible transit of immune cells from the dura to the CNS parenchyma remains unclear, different interchanges between dura and SAS can occur and remain an active area of study (97). Recent investigations have highlighted direct venous connections allowing neutrophils and potentially other myeloid cells to transit between the brain dural vasculature and the skull bone marrow (117, 118).

Dural resident macrophages are characterized as a dense Lyve1^low^MHCII^high^ population, with few Lyve1^high^MHCII^+^ cells present (15) in a different relative proportion compared to the SAS (119). These cells dynamically surveil the local environment while sensing distal gut biome changes (16). Displaying a mixed embryonic and bone marrow origin (6), dural macrophages account for the vast majority of blood-derived myeloid elements found in CNS preparations during homeostasis, together with CCR2^+^ macrophages within the ChP stroma (26). During inflammation, further blood-borne monocytes are locally recruited (97), while dural macrophages can regulate lymphoangiogenesis through the release of VEGF-C (120).

### Resident Choroid Plexus Macrophages

ChPs are located within the third, fourth, and lateral ventricles of the brain and host a functional barrier for immune cell trafficking, the BCSFB. Separating peripheral circulation from the CSF, this barrier consists of a monolayer of epithelial cells connected through tight and adherens junctions (13) and expressing regulatory factors such as macrophage migration inhibitor factor (MIF) (121). On the basolateral side of this layer, a basement membrane and a thin stroma divide the BCSFB from fenestrated blood vessels (122, 123).

Producing the CSF and maintaining its chemical balance, the ChP has been considered as “the kidney of the CNS”, indispensable for homeostatic equilibrium (124–126). Furthermore, the ChP plays roles in brain development, neurogenesis, metabolism (108, 127, 128) and secretes immunomodulatory microRNAs (129). The CSF itself has mechanical and signaling roles exerted through bioactive molecules and physical/chemical properties such as pH, osmolarity, and flow speed (130).

Different macrophages populate the ChP, albeit at a lower density compared to other CNS interfaces (26). ChP macrophages have been historically described as stromal phagolysosome-rich CD163^+^MHCII^+^ antigen presenters (38, 39). Recent studies, however, indicate that the ChP hosts a highly heterogeneous population of yolk sac-derived long-lived stromal macrophages (ChPMs), CCR2^+^ blood-borne macrophages, and Sall1^+^ Kolmer/epiplexus cells situated on the apical side of epithelial cells and thus beyond the BCSFB (16).

The dynamic movement of ChP macrophages has been recently described by *in vivo* two-photon imaging following deep-brain cannula implantation: while epiplexus cells display different kinetic patterns on the apical side of epithelial cells, stromal macrophages continuously surveil ChP vasculature with highly motile processes, efficiently phagocytosing blood-borne fluorescent dextran (131).

Unique among BAMs, epiplexus macrophages share ontogeny, local self-renewal upon depletion, and transcriptome with parenchymal microglia (16). Analysis of the ChP *via* scRNA-seq identified three macrophage clusters sharing high expression of BAM signature genes *Mrc1*, *Ms4a7*, *Pf4*, *Stab1*, *Cbr2*, and *Fcrls* (27). Another scRNA-seq study also described three ChP clusters sharing signature expression of *Lilra5* and *Ttr* and identified as *Cst7^+^Gm1673^+^ Clec7a^+^* epiplexus cells, MHCII^high^ and MHCII^low^ ChPMs, the latter two likely corresponding to yolk sac- and bone marrow-derived resident ChPMs, respectively (16). In parallel, mass cytometry indicates equal numbers of Lyve1^+^MHCII ^negative^, Lyve1^negative^MHCII^+^, and Lyve1^+^MHCII^+^ ChP macrophages, in a proportion which differs from the one observed at other CNS barriers (15).

Interestingly, MHCII expression in ChP macrophages is affected by microbiome alterations likely sensed *via* proximal fenestrated capillaries (16). Unfortunately, the effect of gut flora alterations has not been convincingly investigated in other BAMs.

## Circulating Monocytes, Border Trespassers Upon Inflammation

Origin, function and classification of blood monocytes have been reviewed elsewhere (32, 132–134). Briefly, following monopoiesis, monocytes are mobilized by a CCL2-dependent mechanism from the bone marrow and from splenic secondary reservoir (135) and enter the circulation displaying a half-life of approximately 1–2 days in mice and of 1–7 days in humans, depending on the cellular subset (22, 136–138). In the mouse, two major types of blood monocytes can be described as Ly6C^high^CCR2^+^CX3CR1^low^ “classical” inflammatory monocytes and Ly6C^low^CCR2^low^CX3CR1^high^ “non-classical” patrolling cells (31), with the latter originating from the former both in lymphoid organs and in the periphery (133). While Ly6C^high^CCR2^+^CX3CR1^low^ cells show fast CCR2-mediated recruitment toward inflamed tissues (139), patrolling monocytes mostly participate in endothelial homeostasis within the lumen (137, 140, 141). In humans, a parallel classification exists with “classical” monocytes characterized as CD14^high^CD16^negative^, non-classical cells as CD14^+^CD16^high^ and transitional intermediate monocytes as CD14^high^CD16^+^ (32, 60, 61). A more complex categorization of monocyte subtypes is, however, possible and advisable both for mice and human studies (142–145).

Despite their population-specific differences, all circulating monocytes express high levels of Csf1R and the receptor for granulocyte-monocyte colony stimulating factor (GM-CSFR), platelet endothelial cell adhesion molecule 1 (PECAM-1), and β2 and αM integrins, among others (32, 132).

Monocytes sense inflammation and damage *via* cytokines, chemoattractants, and damage-associated molecular patterns (DAMPs) which contribute to their tissue recruitment (146), with extravasation leading to differentiation to monocyte-derived macrophages (MdMs) (147). Depending on the specific context and highlighting their plastic potential, monocytes can, however, also differentiate into monocyte-derived DCs (148–150) or even to other cellular fates (151).

Dynamic interaction with endothelial cells in the vascular lumen involves a selectin-dependent rolling, a chemokine-dependent arrest and adhesion, and an integrin-mediated crawling eventually resulting into diapedesis (152). Extracellular matrix molecules such as heparane sulfate proteoglycans expressed by the CNS vasculature can also mediate monocyte interaction with endothelial cells (153). Given a differential expression of interaction molecules and chemokine receptors, monocyte subtypes display intrinsic variance in this multistep process (132). Cell deformability through cytoskeletal reorganization and membrane stiffness changes are also regulators of trafficking (154). During trans-endothelial migration, monocytes interact with the endothelial molecules CD99, PECAM1 and CD155 (155) and, following diapedesis, cross the vascular basement membrane and interact with other perivascular cells (144, 156).

Within inflamed tissues, MdMs display substantial differences compared to monocytes. Upregulation of cell differentiation and trafficking genes starts during the first luminal contact with endothelial cells (144, 157, 158), with transmigrated monocytes showing significant changes in metabolism, chemotaxis, survival, inflammatory response (159), and rearrangement in subcellular structures leading to an augmented size (134). Altogether, through the recruitment process, monocytes can acquire distinct pro- or anti-inflammatory polarizations, substantially contributing to pathogen eradication/tissue destruction or to the regulation of inflammation/promotion of tissue regeneration, respectively.

## Macrophage Pro- and Anti-Inflammatory Functions

The acquisition of a functional phenotype by tissue macrophages and MdMs is a highly dynamic process which integrates several local cues and thus remains challenging to define *in vivo*. While these functional adaptations can be modeled and described in high detail *in vitro* (160) through a variety of techniques (161), the signaling pathways and functional activations observed *in vitro* and *in vivo* may diverge significantly depending on the model and the context (162).

Macrophage gene expression displays an inherent plasticity influenced by local signaling, chemical changes and physical confinement (163, 164). While pro-inflammatory macrophages mainly contribute to damage and neurotoxicity by the secretion of chemokines, inflammatory cytokines, and reactive oxygen and nitrogen species, anti-inflammatory cells extensively contribute to neuroprotection by debris scavenging and by releasing tissue regeneration intermediates and growth factors. Functional specifications are also reflected by divergent metabolic adaptations, with pro- and anti-inflammatory polarizations distinctively characterized by differential ATP production and oxygen consumption rates (165). Notably, the acquisition of a specific macrophage phenotype varies substantially also between different mouse strains (166).

To describe the spectrum of macrophage functions, researchers have largely made use of the M1/M2 dichotomy, a jargon introduced in the 1990s to indicate the outcomes of cellular stimulation with IL-4 or lipopolysaccharide (LPS)/IFNγ, respectively (167). Unfortunately, the application of the binary M1/M2 nomenclature to extremely diverse *in vitro* and *in vivo* contexts was unable to properly define multifaceted cellular actions (162, 163, 168). The limitations of this dichotomy were also evidenced when studying microglia/macrophage activation in several pathological contexts, including traumatic and neurodegenerative diseases and disease models (165). While some efforts of clarification in macrophage nomenclature have been made (25, 168–170), a generally accepted consensus is still missing. As suggested by experts in the field (170), we support a jargon describing cellular phenotypes *via* the *in vitro* stimuli used or, in complex *in vivo* scenarios, *via* the observed pro- or anti-inflammatory roles of the described populations.

Besides nomenclature issues, however, differentially polarized macrophage and monocyte subsets from mice and humans possess distinct migratory properties, for example, toward plasminogen (171). Notably, their CNS-invading trajectories and the anatomical site in which they acquire their differential function remain an undeveloped area of study.

## Monocyte Trafficking Through the CNS at Steady State

While accumulation of peripheral immune cells at CNS borders is a hallmark of CNS diseases (172), rapid recruitment of monocytes to perivascular CNS spaces is also observed upon peripheral inflammation, such as in endotoxemia (173). While this highlights the potential for active CNS surveillance by blood-borne myeloid cells notwithstanding the absence of local damage, CNS interfaces at steady state host only a limited number of bone marrow-derived immune cells (89, 174, 175). Recruitment of these cells drastically depends on local tissue accessibility, with interfaces such as the dura mater and the ChP hosting fenestrated vessels and a concomitant higher density of monocytes (16).

Importantly, stromal accumulation of blood-borne leukocytes in the ChP might serve as an intermediate step for reaching the CSF by crossing the BCSFB (176, 177). Analysis of human CSF indicates that approximately 1/3 of the cellular compartment comprises monocytes (178), with a vast majority of blood-borne CD16^high^ cells (68). The homeostatic recruitment of these cells, potentially extravasating at the ChP or directly through leptomeningeal vessels as shown upon CNS damage (47, 112), is, however, unknown.

Given the secluded intraventricular location of ChPs, *in vitro* models have contributed significantly to our understanding of local cell trafficking (179). Using primary ChP mouse epithelial cells, we recently showed that MdMs can migrate through the BCSFB epithelium also in absence of inflammation (37). This transmigration pathway seems possible also for other myeloid cells (180).

Within the CNS parenchyma, basal immunesurveillance is exerted by microglia and pvMs, without apparent contributions by MdMs (14). The ability of MdMs to surveil these border areas at steady state has been historically overestimated due to the absence of tools discriminating yolk sac- and bone marrow-derived myeloid cells and due to the experimental use of chemotherapy or gamma irradiation, artificially increasing BBB permeability and CNS chemokine production (15, 16, 22).

In general, the concept of peripheral immunesurveillance implies that patrolling antigen-presenting cells scan their target organ and, upon infection, move toward secondary lymphoid organs to trigger antigen-specific lymphocyte activation. Key to its relative immune privilege, however, the CNS shows limited afferent routes for cell-mediated antigen drainage (1). Antigen-rich CSF drains to peripheral venous blood *via* arachnoid villi and granulations and to the lymphatic system along nerve roots and nasal and dura lymphatics (12, 181). Notably, the relative importance of these pathways is still under debate (181, 182). Through these exit routes, CNS antigens can accumulate in peripheral lymph nodes (182, 183), potentially *via* DCs trafficking from CNS borders to peripheral organs (28, 184). Whether monocytes and MdMs can also participate in this afferent arm of CNS immunity in a comparable manner to that observed in peripheral tissues (185) is, however, unclear (175).

## Myeloid Dwellers and Trespassers at CNS Interfaces Upon Auto-Aggressive CNS Inflammation

Macrophages constitute the predominant cell type in the damaged CNS of multiple sclerosis (MS) patients, independently from clinical course (169) and lesion subtype (186, 187). Accordingly, MS disease-modifying therapies strongly affect monocyte/macrophage functions as part of their therapeutic action (169, 188–190).

MS is a chronic inflammatory disease of the CNS with unknown etiology and a heterogeneous pathological course, including relapsing-remitting (RRMS), primary and secondary progressive forms (191). Histopathologically, MS is characterized by multifocal BBB damage and leukocyte infiltration in lesions displaying demyelination and neuronal death (192, 193). To date, whether neurodegeneration is the primary cause or rather the secondary consequence of auto-aggressive inflammation remains debated (194).

Blood monocytes isolated from MS patients show altered expression of microRNAs (195), microvescicle release (196), cytokines (197), norepinephrine (198), and enhanced CCL2-, CCL5-, and CXCL1-driven migration (188, 199) compared to cells from healthy controls. The relative proportions of circulating classical, intermediate, and nonclassical monocytes varies across studies, with some indicating a substantial increase in nonclassical CD14^+^CD16^high^ monocytes (65), while recent work shows an increase in CD14^high^ and CD16^high^ monocytes specifically in RRMS patients with inactive disease (66).

Within the CNS parenchyma, resident and invading macrophages play complex roles both preclinically and in established lesions (71). Monocyte invasion might, however, vary at different disease stages, with less MdM infiltrates observed in progressive MS compared to RRMS (200).

Inflammatory macrophage functions range from tissue destruction (103) to beneficial roles (201, 202), a continuum reflecting their unique transcriptional plasticity (163, 170). While microglia actions during MS fall in the same context-dependent classification, slowly expanding lesions from progressive MS patients display high density of pro-inflammatory markers in perilesional microglia, showing how these cells can contribute to disease progression (203). In general, however, it remains unclear whether distinct microglia/macrophage actions are preferentially associated with different phases of lesion evolution, or whether they co-exist at every clinical timepoint or even within the same cells (72, 169).

Albeit heterogeneous, the distribution of MS lesions often follows an expected pattern (204), potentially shaped by routes of leukocyte entry and local antigen presentation (205).

To mimic the multifaceted pathological aspects of MS, several inducible and spontaneous animal models have been established. Among these, experimental autoimmune encephalomyelitis (EAE) has been the main tool to study disease mechanisms and to develop and test MS disease-modifying therapies (169), despite its intrinsic limitations as an MS model (206).

**Figure 2:**
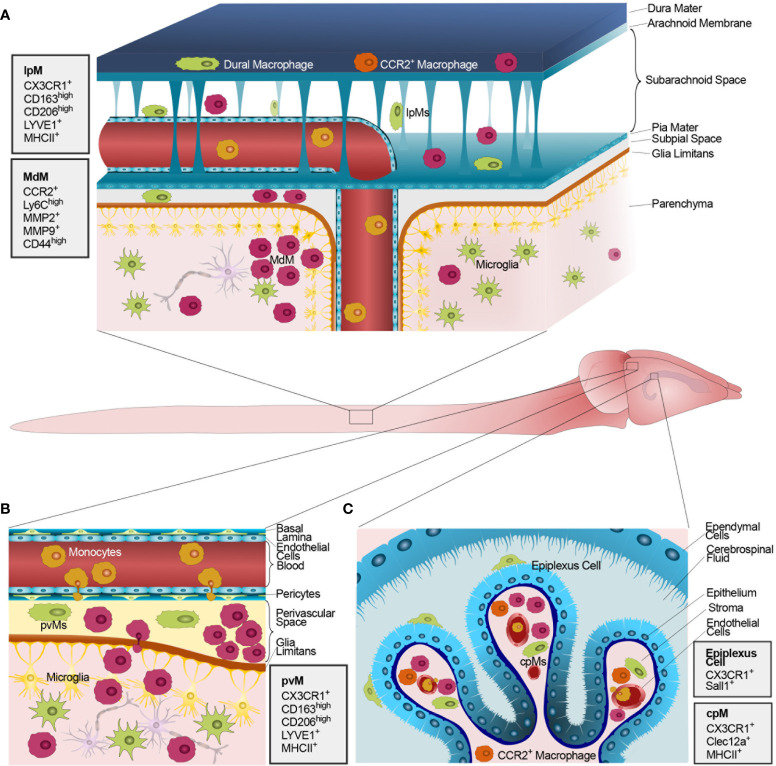
Macrophages populating CNS barriers and parenchyma during autoimmune CNS inflammation. The figure shows the mouse CNS following induction of EAE and disease development. The magnified inlets illustrate schematic representations of the anatomical CNS interfaces containing functional barriers and blood-borne MdMs. **(A)** The mouse meninges. Top to bottom, the dura mater hosts yolk sac-derived (green), blood-borne CCR2^+^ macrophages (orange), and several activated MdMs (red). Different monocytes/MdMs (red) infiltrate the SAS and the subpial space either extravasating at the level of leptomeningeal vessels or crossing the distal ChP BCSFB, thus reaching the CSF. MdMs can invade the CNS parenchyma while yolk sac-derived microglia (green) increase in number. **(B)** Schematic representation of the perivascular space at the level of post-capillary CNS venules, hosting yolk sac-derived pvMs (green) and invading MdMs (red) collectively forming a perivascular inflammatory cuff. After crossing the glia limitans, MdMs (red) accumulate in parenchymal lesions. **(C)** Schematic representation of the ChP within a CSF-filled brain ventricle lined by ependymal cells. The ChP stroma is enlarged compared to steady state and hosts a combination of yolk sac-derived cpMs (green) and different populations of blood-borne inflammatory CCR2^+^ macrophages (orange/red) increasingly extravasated from stromal vessels lacking a BBB. Monocytes circulating within vascular lumens are shown in yellow. The epithelial cells of the BCSFB show decreased density of apical microvilli and are represented as darker and lighter cells to model the ultrastructural alterations previously reported (207). Next to each panel, gray boxes illustrate the main protein markers identifying CNS-resident macrophages in the distinct anatomical compartments, and the main markers commonly expressed by MdMs during CNS inflammation.

As in MS, inflammation in EAE is characterized by a high density of activated macrophages at CNS interfaces and within parenchymal lesions (**Figure 2**) (44). Given the overlapping expression of key markers including CD11b, Csf1R, CD163, and CD206 (21), the relative pathological contributions of MdMs and resident macrophages has remained unaddressed for decades, but technical advancements finally allow us to define their respective roles (169). Upon induction of EAE, BAMs increase their expression of MHCII, CD44, the immunomodulatory molecule PDL1, CD117 (c-KIT), and Sca-1 (Ly6a) (15). Despite convergent morphological and expression changes, resident macrophages and MdMs remain transcriptionally separate (208) and can be distinguished through mass cytometry (15) and scRNAseq techniques (27). The survival dynamics of recruited MdMs remain, however, unclear, with previous work indicating an inability of invading macrophages to persist as microglia-like cells (209) and recent reports showing the opposite (21, 210, 211).

During the active phase of EAE, MdMs substantially outnumber BAMs at CNS interfaces (15). Compared to circulating monocytes, CNS-recruited MdMs upregulate glycolytic enzymes and production of inflammatory molecules (44). While experimental MdM removal by apoptosis reduces disease burden (212), MdM accumulation directly correlates with neurodegeneration (208), likely through an increased production of reactive nitrogen and oxygen species (ROS/RNS) (213, 214). Altogether, monocyte infiltration into the CNS parenchyma parallels EAE clinical signs and exerts a significant influence on glial cells (215, 216), at the same time contributing to tissue regeneration (217, 218).

The overall complexity of macrophage phenotypes during EAE is exemplified by the differential expression of the enzymes iNOS and arginase-1 in CCR2^+^Ly6C^high^ MdMs (44). While iNOS^+^ MdMs revealed higher glycolytic rates, expression of matrix metalloproteinases (MMPs), chemokines (e.g., *Ccl5*), and pro-inflammatory cytokines, arginase-1^+^ MdMs showed increased expression of scavenging receptors, complement proteins and oxidative phosphorylation enzymes (44). Notably and beside iNOS^+^ and arginase-1^+^ cells, a recent study described two Saa3^+^ and Cxcl10^+^ monocyte populations substantially contributing to tissue damage within the CNS parenchyma (43).

Before reaching the white or gray matter, MdMs are increasingly recruited to the ChP stroma (37) and need to cross CNS barriers as shown by accumulation within perivascular cuffs at the BBB (45, 46) or in the CSF, extravasating from the ChP and/or from leptomeningeal vessels. Notably, massive monocyte/macrophage accumulation at CNS borders does not directly lead to clinical symptoms in absence of parenchymal infiltration (219). Passage through these interfaces allows, however, monocytes to adapt to the border microenvironment, acquire distinct functional polarizations and, in turn, regulate the evolution of the disease. Hence, the description of MdM migratory routes and the definition of the parallel role of BAMs represent a fundamental milestone in our understanding of auto-aggressive CNS inflammation.

### Macrophages at the BBB During MS and EAE

BAMs efficiently sense the peripheral environment *via* bidirectional communication with their milieu and in particular with endothelial cells (220), scanning for potential distal danger and reacting to it promptly (100, 221). Experimental evidences from both MS and EAE indeed suggest that pvMs become activated even before development of clinical symptoms and infiltration by peripheral cells (222, 223). Accordingly, pre-clinical CNS lesions observed in marmoset EAE models correlate with increased Iba1^+^ pvMs (224). CNS inflammation leads to a sudden increase in pvM density and to augmented antigen-presenting capabilities (225). These pvMs also increase in number in EAE (42, 226) *via* a local proliferation lasting until the chronic disease phase (14). The observed proliferation rate is, however, minor compared to the one described for microglia (27).

Nearby MS lesions, pvMs express CD68, CD64, CD40, CD32, and MHCII, as well as the signature proteins CD163 and CD206 (71, 72). During their activation, pvMs also upregulate expression of interacting molecules such as ICAM-1 and VCAM-1 and chemoattractants such as CCL2/MCP-1 and CCL3/MIP-1α (42). Notably, in both EAE and MS, pvMs appear highly phagocytic and take up substantial amounts of iron, directly linked to demyelination (227, 228).

Surprisingly, however, scRNA-seq analysis indicates that pvMs undergo only mild modifications to their overall transcriptional profile during EAE, compared to their steady state (27). Among the few significantly regulated genes, an increased expression of *Ccl5*, a T cell chemoattractant, of the MIF receptor *Cd74* (41) and a decreased expression of *Lyve1* and *Ctsd* (Cathepsin D, a bactericidal protein) was observed (27).

In parallel, neuroinflammation correlates with massive recruitment of peripheral monocytes which cross the BBB drastically increasing the cellularity of perivascular spaces. Monocyte mobilization from peripheral reservoirs is regulated by several factors including GM-CSF (149), a cytokine playing key roles in both EAE and MS (229).

MdM recruitment results in the formation of perivascular cuffs, a MS pathological hallmark where lymphocytes intersperse with a majority of CD16^high^ myelin-laden MdMs (208, 230). At least in EAE, perivascular MdMs appear morphologically smaller than activated resident pvMs (27). A series of DAMPs/alarmins including HGMB1, IL33, and ATP participate in the recruitment of monocytes (146). In turn, both *in vitro* (68) and *in vivo* data (45) indicate that MdM perivascular accumulation enhance recruitment and parenchymal invasion of lymphocytes.

Perivascular inflammatory cell cuff formation is often associated with BBB disruption, a multifaceted concept entailing exogenous and endogenous mechanisms (231). Even though BBB damage aid monocyte extravasation, immune cells can cross endothelial barriers showing intact intercellular tight junctions (3, 37, 190). Monocyte diapedesis is aided by the release of inflammatory molecules such as tissue transglutaminase 2, oncostatin M, histamine, superoxide, GM-CSF, and TNFα (232–235). Contact with endothelial cells involves interactions between monocyte integrins such as VLA-4/LFA-1 and endothelial integrin-binding molecules such as VCAM-1/ICAM-1 (169), homophilic interactions through Ninjurin1 (236) and expression of the constitutively expressed adhesion molecule CD166 (237). Mechanistically, contact of monocytes with the BBB allows release of tissue plasminogen activator from endothelial cells following activation of the NMDA receptor NR1, allowing, in turn, monocyte diapedesis (238, 239). Perivascular cell cuff formation is also dependent on the local expression and accumulation of chemokines, including CCL2 (240), CCL3, CXCL12 (46), and potentially of the constitutively expressed CCL19 (241).

In particular, CCL2 actions have been extensively studied in MS and EAE (242). This chemokine can exert distinct roles depending on its astrocytic or endothelial source (240). CCL2 regulates CCR2^+^ monocyte adherence and chemotaxis by acting on monocyte integrin conformation and clustering (243–246). Following tissue invasion, CCR2 expression is downregulated contributing to CNS retention of inflammatory MdMs as observed *in vivo* (44) and *in vitro* (37). Signaling through CCR1 and CCR5 can similarly regulate monocyte multistep accumulation in the perivascular spaces, collectively guiding disease development (185, 247).

Despite its intrinsic pathological consequences, the accumulation of MdMs in perivascular cuffs also constitutes an efficient checkpoint mechanism ensuring that cells do not indiscriminately enter in contact with neuronal cells. To infiltrate the CNS parenchyma from the perivascular space, immune cells need to additionally cross the parenchymal basement membrane and the glia limitans (45). Notably, during neuroinflammation, BBB disruption induces expression of tight junctions in astrocytic end-feet in both EAE (248) and MS (249). Crossing of this second barrier crucially requires expression of MMPs and of MMP regulatory proteins such as CD147 (169). In this process, MMP2 and MMP9 participate in the lysis of perivascular chemokines such as CXCL12, that increase retention of MdMs preventing parenchymal infiltration (45, 46).

Are perivascular MdMs functionally polarized during anti-CNS responses? While interacting with endothelial cells, transmigrating monocytes encounter several activation signals. GM-CSF, shown to augment monocyte diapedesis at the BBB, can induce a hybrid inflammatory phenotype similar to the one observed in MS tissues (232). GM-CSF can also be released by endothelial cells (250, 251) upon stimulation by Il-1β, an inflammatory cytokine released by invading monocytes and indispensable for their infiltration (251), in a looping mechanism potentiating MdM activation between BBB and parenchyma (252). Furthermore, feedback regulation by reactive species-mediated quorum-sensing might play a key role in phenotype adaptation (30). MdMs within the perivascular space can have an iNOS^+^ or Arginase-1^+^ phenotype; furthermore, *in vivo* imaging indicated that MdMs acquired a pro-inflammatory state immediately following diapedesis and before entering the CNS parenchyma (44). Accordingly, perivascular accumulation of glycolytic phagocytes has been observed in both EAE and MS, with recruited MdMs reducing their rate of glycolysis once in the parenchyma (253). Inhibition of glycolysis and of lactate secretion reduced macrophage invasion, both *in vivo* and *in vitro* (253). MdMs in perivascular cuffs were strongly positive for *Nrf2*, a transcription factor involved in protection against oxidative stress and highly expressed in acute MS lesions (73). Moreover, in both MS and EAE, these cells upregulate expression of the extracellular matrix components lectican versican V1 and chondroitin sulfate glycosaminoglycans, molecules able to enhance MdM migration and secretion of inflammatory cytokine and chemokines (254). Finally, pvMs in active demyelinating MS lesions also show strong reactivity for TGFβ (255), an anti-inflammatory molecule with controversial roles (256).

Taken together, the perivascular space thus appears like a key compartment able to shape the pathological role of recruited monocytes in their migration toward the inflamed parenchyma.

### Macrophages in the SAS During MS and EAE

Meningeal inflammation is common in MS clinical manifestations, including primary progressive forms (67, 257), often correlating with neurodegeneration (258). The meninges of MS patients can also host lymphoid follicle-like structures rich in B lymphocytes, potential drivers of disease (259).

However, histological analysis reveals that CD68^+^ myelin-laden phagocytes are the most represented cell type in the inflamed SAS (67). Collection of CSF *via* lumbar puncture allows analysis of non-adherent SAS phagocytes and indicates significant variations in CSF cellularity depending on the MS subtype. Compared to healthy donors, the CSF of RRMS patients shows a relative decrease in CD16^+^ monocytes and an increasing proportion of CCR5^high^CD64^+^CD86^+^CD14^+^ monocytic cells (68). The described decrease in CD16^+^ monocytes is not observed in primary progressive patients, potentially reflecting different recruitment mechanisms (68). Other studies have shown an increased presence of monocytes expressing HLA-G, an immunosuppressive non-classical MHC molecule (69). Notably, a recent scRNA-seq analysis of the CSF of MS patients identified a majority of monocytes expressing HLA-DR and the lectin CD33 and a small population of HLA-DR^high^CD33^mid^Lyve1^+^ macrophages identified by the authors as “microglia” due to their expression of *Trem2* and *Olr1* (70). This jargon is, however, misleading, as no evidence of parenchymal microglia crossing the glia limitans toward the CSF exists. In another study, a comparable population (named “Mono2”) showed expression of BAM markers such as *Stab1* and *Ch25h* and of inflammatory genes such as *Cd9*, *Cd163*, *Egr1*, *Btg2*, *C1qa*, *C1qb*, *Maf*, and *Csf1R* (62).

Notwithstanding their controversial classification, SAS lpMs clearly play a key role in MS/EAE by producing inflammatory molecules such as CCL5, CXCL9, CXCL10, and CXCL11, in turn, recruiting further leukocytes into the CSF (112, 260).

In EAE, lpMs increase their Iba1 immune reactivity several days before clinical onset of disease, thus indicating a role in the initiation of local inflammation (40). LpMs are highly dynamic dwellers and interact extensively with invading T cells, increasing their proliferation rate during the acute EAE phase (27, 112). This increase in number drops during the chronic phase of disease, paralleled by local appearance of apoptotic lpMs (27). Notably, similar to pvMs, lpM activation in EAE does not lead to dramatic transcriptome changes compared to homeostatic conditions (27).

In MS and EAE, monocytes/MdMs also accumulate in large numbers in the SAS following extravasation from the leptomeningeal vasculature or from the ChP and CSF-filled ventricles. Infiltration through leptomeningeal vessels follows increased intraluminal monocyte crawling and expression of the enzyme tissue transglutaminase 2 (261), known for its involvement in cell adhesion to fibronectin, a glycoprotein released by endothelial cells and pericytes (262).

MdM and lpM dynamics in the SAS during EAE have been extensively explored by intravital imaging (223). Preclinically, lpMs cluster around leptomeningeal vessels following leakage of plasma fibrinogen, which, in turn, triggers ROS production (222). Studies in rats have demonstrated that meningeal phagocytes can present both self and non-self antigens and thus activate infiltrating T cells in a multistep process requiring chemokine signaling (112, 263–265).

Long-lasting contacts seem to occur preferentially between lymphocytes and blood-borne CCR2^+^ monocyte-derived cells, rather than with resident BAMs (27).

Surprisingly, however, meningeal macrophages do to play an essential role in antigen presentation during EAE. A series of recent reports convincingly demonstrated that expression of MHCII in CD11c^+^ classic DCs but not in CX3CR1^+^ macrophages is indispensable for disease induction (28, 266, 267). Nonetheless, the frequency of lpM and MdM contacting lymphocytes suggests the existence of further regulatory roles shaping EAE. In our work, the majority of SAS MdM displayed strong expression of arginase-1^+^ in striking contrast to the iNOS^+^ dominated nature of parenchymal lesions, potentially indicating an anti-inflammatory function of lpMs (44). The differential representation of MdM phenotypes in the SAS and in other CNS compartments might also be related to distinct sensitivity toward chemoattractants, as shown *in vitro* using differentially polarized human cells (268, 269), either as a result of differential chemokine receptor expression or a differential receptor response to transduction.

From the SAS, activated MdMs can reach the CNS parenchyma and participate in the formation of subpial demyelinating lesions, an histological hallmark of progressive MS forms (191). The contribution of MdMs, however, depends on the type of lesion, with so-called leukocortical plaques showing a high number of activated macrophages and purely subpial cortical lesions mostly devoid of inflammatory infiltrates (257). In EAE, subpial white matter demyelination is commonly described in the spinal cord, but cortical gray matter pathology, as observed in MS, is rare. The latter type of lesion can, however, be modeled in mice through cortical injections of TNF and IFNγ (270, 271) or by peripheral injection of β-synuclein-specific T cells (272).

To reach the CNS parenchyma from the SAS, meningeal MdMs need to transverse the pia mater, the parenchyma-associated basement membrane and, eventually, the glia limitans (10). *In vivo* imaging has shown that cells within the SAS might move toward the parenchyma by crawling on the external surface of leptomeningeal vessels entering the parenchyma (273). However, the permeability of the pia mater to immune cell trafficking remains debated, and the required interaction molecules are unknown (104, 107, 260).

Migration of MdMs from the meninges to the parenchyma can be downregulated by the administration of CXCR7 antagonists, impeding CXCL12 signaling and resulting in meningeal accumulation (274). Retention of phagocytes in the SAS was paralleled by loss of VCAM-1 on astrocytes, thus highlighting a potential role of these cells as interactive partners in the invasion process (275).

To summarize, even though anatomical differences between the meningeal system in rodents and humans impede a fully coherent discussion, several reports have evidenced the central part played by leptomeninges in initiation and evolution of autoimmune CNS inflammation. Nonetheless, many unsolved questions exist regarding macrophage functions and trafficking routes. A detailed anatomical description of these compartments and the creation of transgenic animals allowing visualization of defined meningeal layers (116) remain crucially needed for the progress of the field.

### Macrophages in the ChP During MS and EAE

In the context of auto-aggressive CNS inflammation, the ChP has been proposed as the first CNS gateway for autoreactive lymphocytes prior to BBB disruption, subsequently triggering a secondary leukocyte CNS infiltration driving disease progression (40, 260, 276).

Rather than a sealed barrier, the BCSFB is considered an active yet highly regulated exchange surface (108) showing a differential expression of tight junctions compared to the BBB (123).

Immune cell trafficking at the BCSFB seems to be regulated by IFNγ-dependent activation in immune surveillance and repair (277). Both CCL20 and CX3CL1 are constitutively expressed at the ChP and might guide recruitment of CCR6^+^ (278) and of CX3CR1^+^ leukocytes, respectively (279). The BCSFB is highly sensitive to systemic inflammation. Thus, peripheral LPS administration leads to local TNF and IL1β secretion, upregulates CXCL1 and CCL2 (280, 281), and triggers release of destabilizing MMP8 and MMP9 (282) and impairment of tight junction barrier properties, an overall reaction suggesting higher trafficking of immune cells.

In the ChP of MS patients, the tight junction protein claudin-3 is downregulated compared to healthy controls (283). Reports of its role in EAE models are, however, controversial, with its deletion increasing numbers of CSF-infiltrated MdMs in one study (283) and to a lack of BCSFB impairment in a recent report (284).

Interestingly, interaction molecules such as ICAM-1 and VCAM-1 are specifically expressed on the apical side of the BCSFB epithelium, facing the CSF (285). During EAE, their increased expression and a *de novo* apical expression of MAdCAM-1 can be observed (207). Notably, while leukocytes crossing the BCSFB toward the CSF utilize ICAM-1 in the last steps of diapedesis (286), the apical location of these molecules seemingly indicates that leukocytes can also migrate backward from the CSF to the ChP stroma (286, 287).

As shown by 2-photon microscopy, ChP macrophages readily respond to peripheral LPS injections by moving toward nearby vessels, with focal ChP damage leading to spatial reorganization of epiplexus cells around the injury site. In both scenarios not all macrophages responded to the danger stimuli, again highlighting the heterogeneity of ChP dwellers (131).

Compared to steady state, induction of EAE leads to the appearance of disease-associated ChPMs (27). These activated cells show significantly increased expression of antigen presentation molecules, chemokines and cytokines such as *Il1b*, with one cluster strongly positive for MHCII genes and for *Ctss* (encoding for Cathepsin S), and the other showing high expression of the antimicrobial products *S100a9*, *S100a8*, and *Ngp* (27). The presence of CCR2^+^ MdMs in the ChP appears substantially enriched throughout the disease, with a minor proportion of these cells locally expressing iNOS and/or arginase-1 (37).

ChP MdM populations also show high CD74 positivity and can be divided in three different cellular clusters composed of Ly6C^high^ monocyte-like, *Cd209^+^* DC-like and *MertK^+^* macrophage-like cells (27).

Beside observations in EAE, not much is known about ChP macrophages in MS. Analysis of human ChP tissue revealed a high density of CD68^+^MHCII^+^ macrophages and a minor proportion of MHCII^negative^ Iba1^+^ cells, with these cells present within the stroma, intercalated between epithelial cells or lying on the apical side of epithelial cells (63, 64). However, the densities of these cells appeared comparable between progressive MS patients and healthy controls (64).

Do MdMs really access the CSF *via* the ChP during autoaggressive neuroinflammation? In non-autoimmune disease models, monocytes/macrophages were indeed shown to cross the BCSFB toward the CSF (47, 288, 289). Using *in vitro* BCSFB models, we could recently confirm that functionally polarized mouse macrophages can actively migrate through the BCSFB monolayer (37). Apparently migrating MHCII^+^CD68^+^ macrophages have also been described interspersed between epithelial cells in the ChP of MS patients (63), yet these cells might represent DC surveillants bridging across the BCSFB (290).

To summarize, while in MS the gateway function of the ChP remains unsupported by direct evidence, an active role of the BCSFB in MdM recruitment to the CNS is highly plausable and this CNS interface should become a focus of attention in neuroinflammatory research.

## Monocyte/Macrophages at CNS Interfaces in Traumatic CNS Injury

Despite shielding by bones, meninges, and CSF, traumatic damage to the CNS parenchyma is a common pathological occurrence leading to neurodegeneration and to an innate immune response promoting further tissue damage (291, 292). Physical insults can occur to the brain (traumatic brain injury, TBI) or to the spinal cord (spinal cord injury, SCI), with these two compartments showing the evolution of distinct pathologies (293). Depending on their severity, mechanical injuries to the CNS result in local death, DAMP release, activation of BAMs and to different degrees of MdM infiltration (146). Interestingly, compared to brain lesions, physical damage to the spinal cord generally results into a higher activation of CNS macrophages, stronger BBB damage, and denser MdM accumulation (293).

### Spinal Cord Injury

Following SCI, perilesional microglia proliferate creating a protective “microglial scar” in concert with astrocytes (294). Communication between microglia and infiltrating MdMs influences their reciprocal polarization as well as lesion evolution (215). While removal of microglia in SCI models proved detrimental, the role of BAMs in SCI was not convincingly addressed. One report showed that pvMs and lpMs do not participate in the disease process, nor do they proliferate extensively in response to SCI (294). The seemingly minor role of BAMs in SCI evolution is supported by observations in demyelinating models: following intra-parenchymal injections of lysophosphatidylcholine, Lyve1^+^ lpMs and pvMs do not penetrate into demyelinated spinal cord lesions (48). Nonetheless, more research is crucially needed to clarify BAM participation in SCI.

Upon injury, spinal cord endothelial cells upregulate the expression of VCAM-1 and ICAM-1 allowing first neutrophils and later monocytes to accumulate in the damaged region (295). Interestingly, these myeloid cells originate mostly from the spleen reservoir pool rather than from the bone marrow, a pattern likely related to the acuteness of the disease (135, 296). Similar to what is observed in EAE models, sensing of CCL2 and CXCL12 and production of MMP9 are required for monocyte migration from the BBB to the parenchyma upon SCI (297, 298). CCL2 and other chemokines released by glial cells such as astrocytes might also contribute to the acquisition of a functional phenotype by invading and local macrophages (299). Local TNF-α production increases MMP-9 expression highlighting a complex interplay of cytokines and proteases. Once in the parenchyma, MdMs follow a gradient of C5a molecules toward the lesion epicenter in a mechanism regulated by IRF8-purinergic receptor axis, all leading to enhanced tissue repair (300). Accordingly, MdMs in SCI have been described as beneficial (146). Conditional ablation of CD11c^+^ phagocytes during the first week after SCI resulted in worsened clinical recovery, however, did not affect the pathology when induced 2 weeks following damage (301). The beneficial effect of MdMs was attributed to their expression of anti-inflammatory IL-10 at the lesion margin (301). Notably, a follow-up work showed that while Ly6C^hi^CX3CR1^lo^CCR2^hi^ monocytes infiltrated the parenchyma in a CCL2-dependent manner *via* leptomeningeal vessels proximal to the lesion, anti-inflammatory Ly6C^lo^CX3CR1^high^CCR2^lo^ cells entered the CNS trafficking through the BCSFB (47). In this study, monocyte migration at the ChP relied on VLA4/VCAM-1 interactions as well as on the expression of the adenosine-catalyzing enzyme CD73. Intriguingly, it has been postulated that these polarization differences could be related to the cellular constituents of the two barriers, epithelial cells for the BCSCF and endothelial cells for leptomeningeal vessels (47).

Nonetheless, the net contribution of MdMs to SCI appears time- and location-dependent (302–305). Indeed, one report described locally recruited MdMs as pro-inflammatory players surrounded by anti-inflammatory microglia distal to the injured area (306). Acquisition of an anti-inflammatory phenotype is also affected by the activation status of nearby astroglial cells (299). In another work, inhibition of monocyte infiltration (via splenectomy) accordingly resulted into an ameliorated clinical phenotype (307). Lastly, other studies have observed that MdMs in the lesioned parenchyma show co-expression of pro- and anti-inflammatory markers, once more highlighting the non-binary role of MdMs in CNS injury and indicating complex local functions upon recruitment (308, 309).

### Traumatic Brain Injury

Similar to SCI, TBI shows a long-term pathological evolution involving excitotoxicity, cytokine release, ROS/RNS production, and infiltrating myeloid cells with neurotoxic as well as neuroprotective functions (310). Beside the extensively investigated role of microglia (50), an involvement of BAMs upon TBI has been suggested by both human and animal studies.

In TBI patients, CD163^+^ microglia/macrophages are increased in both the lesion and perivascular spaces indicating a potential participation of CD163^+^ pvMs to damage evolution (74). CD163^+^ cells are also increased in a rat TBI model two days post TBI, however, mainly within the lesion (49). Importantly, most CD163^+^ cells co-expressed heme oxygenase-1 (49), a key enzyme in heme catabolism (311) exerting anti-inflammatory effects (312, 313) likely promoting neuroprotection following TBI. Nonetheless, in these studies, the peripheral or resident nature of pvMs was not convincingly defined.

Blood-borne MdMs play instead a recognized and context-dependent role in TBI (314). Pro-inflammatory macrophages are recruited early in the lesioned area, with CCR2^+^ MdMs following gradients of chemokines released from activated parenchymal cells (50). Notably, CCR2^+^ monocytes seem to mediate local ROS/RNS production (315, 316) and might thus constitute important pharmacological targets. Indeed, intravenous injection of immunomodulatory nanoparticles reduced MdM recruitment by affecting monocyte survival and sequestration within the spleen (190), altogether leading to a strong reduction of lesion volume (317).

Conversely, other studies have provided evidence for a beneficial effect of monocyte recruitment after mild TBI, for instance by reducing meningeal vascular damage (318), a pathological hallmark associated to peripheral immune response (319). In the latter study, while “classical” debris-scavenging monocytes were located at the center of meningeal lesions, wound-healing “non-classical” monocytes were localized peri-lesionally and promoted meningeal angiogenesis *via* expression of MMP-2 (318).

How do MdMs access the CNS parenchyma following TBI? Analysis of patient tissues suggests that CD14^+^ monocytes initially migrate toward the perilesional perivascular space within the first days following damage and then move toward the parenchyma (75). These MdMs can remain in the perilesional area for weeks (75). In a rat model of TBI, monocytes were instead shown to enter the lesioned CNS parenchyma by crossing SAS microvessels, subsequently accumulating near the injury site (320). Some MdMs appeared to move a short distance along perivascular spaces toward the brain parenchyma (320). This trafficking route utilized by MdMs (and neutrophils) requires cellular interactions that can also be mediated by JAM-A, a junctional adhesion molecule also expressed by macrophages (321, 322).

A potential role of the ChP as a monocyte access gateway in TBI models was suggested by the rapid increase in CCL2 production by the ChP ipsilateral to the lesion. The resulting rise in CCL2 CSF concentration mirrors observations in the CSF of severe TBI patients (323). BCSFB epithelial cultures indicated that CCL2 is secreted across both the apical and basolateral side, a bidirectional production necessary for leukocyte migration at the BCSFB following TBI (288). Accordingly, the blocking of this signaling reduced CCR2^+^ monocyte infiltration and lesion volume (324) and improved neurological recovery after TBI (315, 323, 325). Interestingly, lack of MdM infiltration correlated with increased astrocyte proliferation and reduced astroglial scar formation, thus suggesting a key role of juxtavascular astrocytes in the interaction with MdMs (326).

Taken together, evidence for a potential involvement of BAMs in traumatic CNS disorders remains sparse. Furthermore, experimental approaches allowing selective investigation of yolk sac-derived macrophages have not yet been utilized in this context. The parallel role of CNS-infiltrating monocytes has been more extensively described and appear extremely dependent on recruitment timing, damage extent and lesion location. A better definition of the role of MdMs would potentially aid the development of novel therapeutics for patients suffering from Sor TBI. and/or TBI.

## Myeloid Dwellers and Trespassers at CNS Interfaces in Neurodegenerative Diseases

### Parkinson’s Disease

Parkinson’s disease (PD) is a progressive neurodegenerative disorder characterized by neuronal loss in the substantia nigra pars compacta (SNpc) and by chronic CNS inflammation in both patients and animal models (327, 328). Histopathologically, brain samples of PD patients show accumulation of Lewy bodies rich in the neuronal protein α-synuclein (329). Post-mortem and imaging analysis of PD patients revealed detrimental microglial activation (330, 331) accompanied by upregulation of iNOS as well as production of pro-inflammatory cytokines within the parenchyma (332–334). Strong to moderate microglial activation is also observable in the different animal models of PD (327, 335, 336).

However, the role of BAMs in disease progression has rarely been addressed. In a viral model of synucleinopathy, degeneration of neurons in the SNpc coincided with local CD206^+^ pvM expansion, similar to what has been observed in post-mortem samples of PD patients (52). Depletion of pvMs and lpMs by clodronate liposomes resulted in a significant loss of dopaminergic neurons within the SNpc after two weeks, suggesting that BAMs exert a neuroprotective function in the pathology (52). Notably, clodronate administration increased vascular expression of VCAM-1, enhancing CNS accumulation of lymphocytes (52). Absence of pvMs led to aggravated spreading of misfolded α-synuclein (52), a pathological hallmark of PD (337). On the other hand, in a 6-hydroxydopamine-mediated PD model, no increase in rod-like CD163^+^ pvMs was found in the striatum as opposed to the recruitment from the blood stream of CD163^+^ “polygonal” cells (53). Notably, these parenchymal CD163^+^ cells are also described in the brain of PD patients in association with Aβ deposition and a damaged BBB (77). BBB impairments are commonly observed in both patients and animal models (338).

Several studies have documented an augmented presence of inflammatory cytokines in the CSF of PD patients (338), the increased expression of MHCII in CSF monocytes (76), the presence of α-synuclein at the BCSFB (282, 339) and a potential beneficial role exerted by ChP epithelial cells in transplantation experiments (340). Beyond this sparse evidence of a role of the ChP and in general of BAMs however, the potential contribution of these cells to PD evolution remains unexplored.

Conversely, several studies addressed monocytes/MdMs functions in PD pathogenesis. The total number of blood monocytes is not affected in PD patients (78), but these cells appear less responsive to activation and more proliferative (341) and show altered phagocytosis (78, 342). Furthermore, monocytes from PD patients display an upregulated FAS/FAS ligand system (78), potentially enhancing myeloid cell recruitment and release of cytokines (343, 344). PD patients show increased CCL2 blood levels and dysregulated CCR2^+^ monocytes responses (78, 79). Transcriptionally, monocytes from PD patients show a specific expression profile correlating with disease severity and indicating enriched expression of genes related to migration and regulation of inflammation (345).

Blood monocytes with pro-inflammatory features are also increased in PD animal models (346) and seem to infiltrate the degenerating substantia nigra by crossing the BBB (347, 348). Accordingly, CCR2^+^ MdMs can be found in the brain of PD mouse models early in the disease process, with astrocytes being the main producer of CCL2 (51). Of note, while blocking monocyte recruitment had no effect on dopaminergic neuron survival, overexpression of CCL2 in astrocytes did increase neuronal death and led to augmented infiltration of CCR2^+^ monocytes, together suggesting that MdMs contribute to neurodegeneration (51). Notably, in the SNpc of a different transgenic PD model, MdMs vastly outnumbered microglia and could be engineered as a “Trojan horse” approach to locally deliver neuroprotective factors (349).

While these studies imply trafficking of monocytes across the BBB during PD, the potential contribution of meningeal and ChP gateways to cell recruitment has never been addressed in the literature. In summary, several evidences point toward an involvement of recruited monocytes to PD pathogenesis, setting the ground for future studies finally testing the importance and the therapeutical value of these cells in PD.

### Alzheimer’s Disease

Alzheimer’s disease (AD) is a neurodegenerative disorder characterized by brain atrophy, synaptic loss, extracellular deposition of amyloid-β (Aβ) peptides and intracellular accumulation of neurofibrillary tangles of phosphorylated Tau protein (349–351). The controversial contribution of microglia to AD pathogenesis is discussed by a growing body of literature, with molecules such as CX3CR1, APOE and triggering receptor expressed by myeloid cells 2 (TREM2) showing a critical impact on disease evolution by regulating phagocytosis and anti-inflammatory signaling in macrophages/microglia (352–355).

In parallel, some studies have highlighted the multifactorial contribution of BAMs to AD. Aβ tends to accumulate with age in insoluble depositions limiting drainage along perivascular pathways and typically resulting into cerebral amyloid angiopathy (104, 356). PvMs importantly participate in clearing perivascular Aβ, as shown in different mouse models of AD in which ablation of pvMs resulted in augmented Aβ accumulation (88, 357). PvMs Aβ clearance depended on expression of the scavenger receptor class B type 1 (55) and of CCR2 (56). These pvMs also express CD36 (57), one of the main receptors for Aβ (36). Notably, some studies have demonstrated a CD36-dependent, *Nox2*-mediated production of ROS in response to Aβ phagomacrocytosis in pvMs, a phenomenon ultimately augmenting vascular pathology and cognitive dysfunctions (358, 359). Taken together, pvMs seem to play both beneficial and detrimental roles in AD models, however, their function in AD patients remains undefined.

The complex equilibrium between fluid drainage and Aβ deposition observed at the perivascular level in AD is also detected within the ChP. Aβ transporter proteins are expressed at both the BBB and BCSFB contributing to a coordinated clearance of Aβ into the peripheral circulation (123, 360, 361). Underscoring disease-specific defects in barrier transport mechanisms, ChP tissue from AD patients shows substantial deposition of Aβ peptide and increased oxidative stress (362). Within the ChP of AD patients, dense fibrillary phosphorylated Tau can also be shown in calcified intracellular inclusion in the proximity of TREM2^+^ stromal ChPMs (58, 80). These events lead to significant changes in BCSFB permeability (130, 363) and to a reduced ChP expression of trafficking and inflammatory molecules including ICAM-1, VCAM-1, CXCL10, CCL2, and IFNγ, suggesting impaired monocyte migration *via* the BCSFB and a exacerbatory impact on disease evolution (364).

Concerning meningeal macrophages, one recent report has suggested a contribution of these BAMs to Aβ pathology, yet only within the dura mater and following experimental ablation of lymphatic vessels leading to local accumulation of Aβ and of Iba1^+^ macrophages (365).

While the role of BAMs in AD remains underinvestigated, the impact of MdM on disease development has been addressed in several studies. *In vitro*, monocyte trafficking at the BBB drastically increased in the presence of Aβ and was mediated by receptor for advanced glycation end products (RAGE) and PECAM-1 expressed on endothelial cells (366). Furthermore, both mouse and human microglia stimulated with Aβ upregulate their expression of CCL2, CCL3, CCL4, and CXCL2, suggesting a substantial Aβ-dependent recruitment of immune cells to diseased brains (367–369). Accordingly, different CD45^high^CD11b^high^CCR2^+^ macrophages accumulate in the brain of animal models of AD, with CCR2 deficiency leading to the detrimental accumulation of Aβ (59, 370). These earlier results indicated that MdMs might exert a beneficial effect in the disease process (370, 371). Accordingly, parenchymal CD163^+^ microglia-like MdMs were described in the brain of AD patients in association with Aβ deposition nearby the damaged BBB (77). Unfortunately, in most of these reports, full body irradiation paradigms were utilized (372), artifactually leading to long-term changes in glia activation and increase in myeloid cell recruitment (56, 373). By shielding the mouse brain during irradiation, it was subsequently shown that CCR2^+^ MdMs only rarely infiltrate the CNS, thus redimensioning their role in AD (56). This work also contradicted earlier results (367) by showing that microglia accumulation close to Aβ does not strictly depend on the CCL2 system (56).

Nevertheless, monocytes might contribute to Aβ removal without leaving the vascular tree. A recent study has illustrated the role of CX3CR1^+^Ly6C^low^ patrolling monocytes in crawling in Aβ^+^ brain veins and engulfing intraluminal Aβ (54). However, contradictory observations have been made in AD patients, showing that circulating monocytes express reduced TLR levels (374), are defective in Aβ phagocytosis (375, 376) and are more prone to apoptosis compared to monocytes isolated from control patients (376).

In conclusion, in both mouse models and AD patients monocyte/MdM functions remain somewhat elusive. Studies on BAMs have, however, highlighted their complex role in Aβ removal and thus potentially suggested these cells as future targets of therapeutical interventions.

## Conclusion and Outlook

Aided by stimulating debates on functional CNS anatomy (13, 97, 106, 182), by technical advancements in reporter tools and imaging (14, 44, 131) and by the recent “single-cell analysis revolution” (16, 27, 28), the study of CNS borders finally bloomed as a research field.

Long-lived dwellers of these CNS interfaces, BAMs mediate systemic communication (4), regulate vascular permeability (91, 92), waste clearance (94), fluid drainage (88), and surveil CSF composition (34). Often ignored and improperly ontogenically and anatomically defined, BAMs can now be precisely identified in contrast to blood-borne myeloid cells (14–16, 20, 22, 23), a necessary advancement allowing to address specific cell functions upon CNS damage. In this context, each CNS interface becomes a complex battleground hosting a myriad of peripherally derived trespassers. Even though the multidimensional interplay between invading monocytes, BAMs and the cellular components of each CNS gateway serves as a key checkpoint in disease evolution, a convincing picture of macrophage dynamics at CNS borders is far from existing.

What is the contribution of BAMs to pathogenesis of CNS disease?

During autoimmune neuroinflammation, BAMs increase in density, contact invading leukocytes and produce inflammatory cytokines and chemokines (42, 112, 225, 260). Surprisingly, however, these cells modify their overall transcriptional profile only mildly compared to steady state (27). Upon TBI/SCI, BAM actions seem beneficial but at the same time negligible (48, 49, 74, 294). The same applies to PD (52, 53, 76), while AD studies conversely indicate a disease-shaping role of BAMs (88, 357, 358, 365). Notably, the discussed results and recent data from brain tumor models (24) seem to indicate a relatively minor contribution of BAMs to the evolution of most CNS dysfunctions.

Conversely, during neuroinflammation, MdMs outnumber BAMs at CNS interfaces and drive disease evolution (15). Molecules such as GM-CSF (149, 229), CCL2 (240, 243–246), and different MMPs (169) primarily orchestrate the overall immigration of these cells into the CNS parenchyma, but the actual route of invasion through different CNS gateways remain remarkably speculative. Monocyte recruitment upon CNS trauma shares similar mechanisms with MS/EAE including the role of CCL2 and the need for MMP production by infiltrating cells (297, 298, 377). Lastly, monocyte trafficking to the CNS during neurodegenerative disorders remain surprisingly understudied, and its dependency on the CCL2-CCR2 axis controversial (51, 56).

To summarize, despite various efforts to understand the functional contribution of myeloid cells to CNS diseases, this review underlines how the study of BAMs and of monocytic invasion pathways is still at its infancy. Recent technical advancements should finally allow understanding whether BAMs, as long-lived dwellers of CNS interfaces, can become relevant therapeutic targets to manipulate CNS dysfunctions. Secondly, a renewed focus on CNS anatomy and barrier functions will hopefully prompt scientists to investigate and describe invading trajectories of monocytes/MdMs in the different disease models. A detailed definition of the infiltration routes and of the polarizing influence of distinct CNS gateways on trespassing monocytes will eventually allow designing targeted strategies to regulate monocyte entry, thus modulating the evolution of CNS pathologies.

## Author Contributions

JS designed the figures. DI prepared the tables. DI, SW, KB, and GL wrote the manuscript. All authors contributed to the article and approved the submitted version.

## Funding

This work is supported by a Swiss Multiple Sclerosis Society grant, Italian MS grant (2019/R-Single/001) and by Scherbarth Foundation funding awarded to GL. 

## Conflict of Interest

The authors declare that the research was conducted in the absence of any commercial or financial relationships that could be construed as a potential conflict of interest.

## Abbreviations

CNS, central nervous system; SAS, subarachnoid space; BCSFB, blood-cerebrospinal fluid barrier; BBB, blood-brain barrier; ChP, choroid plexus; lpM, resident leptomeningeal macrophage; pvM, resident perivascular macrophage; ChPM, resident stromal choroid plexus macrophage; MdM, monocyte-derived macrophage; BAM, barrier associated macrophage; MS, multiple sclerosis; EAE, experimental autoimmune encephalomyelitis; ROS/RNS, reactive oxygen and nitrogen species; SCI, spinal cord injury; TBI, traumatic brain injury; AD, Alzheimer’s disease; Aβ, amyloid-β; PD, Parkinson’s disease; DAMPs, damage-associated molecular patterns.
